# Dietary copper intake and risk of myocardial infarction in US adults: A propensity score-matched analysis

**DOI:** 10.3389/fcvm.2022.942000

**Published:** 2022-11-10

**Authors:** He Wen, Xiaona Niu, Lang Hu, Nan Sun, Ran Zhao, Qiuhe Wang, Yan Li

**Affiliations:** Department of Cardiology, Tangdu Hospital, The Second Affiliated Hospital of Air Force Military Medical University, Xi’an, China

**Keywords:** dietary copper intake, myocardial infarction, health risk, NHANES, propensity score matching

## Abstract

**Objectives:**

Most studies have examined the association between serum copper and myocardial infarction, but there is little evidence of the association between dietary copper intake and myocardial infarction.

**Materials and methods:**

The study included a total of 14,876 participants from the 2011 to 2018 National Health and Nutrition Examination Survey (NHANES). Multivariate logistic regression model was used to analyze the association between dietary copper intake and the risk of myocardial infarction. To reduce selection bias, we use nearest neighbor propensity score matching (PSM) in a 1:2 ratio. Restricted cubic spline (RCS) method is used to study the non-linear relationship. Subgroup stratification was used to further investigate the association between copper intake and myocardial infarction.

**Results:**

The median dietary copper intake was 1.0825 mg/day. A myocardial infarction had occurred in approximately 4.4% (655) of the participants. Before and after matching, multivariate logistic regression models revealed a negative correlation between dietary copper intake and the risk of myocardial infarction. The higher quartile of subjects had a noticeably lower risk of myocardial infarction in comparison to those in the first quartile of copper intake. According to RCS findings, dietary copper intake and myocardial infarction have a non-linear and dose-response relationship. According to stratified analysis, the dietary copper intake was a substantial protective element for those who were ≥ 50 years old, female, 25 ≤BMI <30, with history of smoking, hypertension, diabetes and ortholiposis.

**Conclusion:**

Increased dietary copper intake was associated with a lower risk of myocardial infarction. It is especially significant in elderly-aged women, overweight individuals, smokers, hypertension, and diabetic patients.

## Introduction

The major cause of mortality and morbidity worldwide is cardiovascular disease (CVD), and myocardial infarction (MI) is one of the most serious and lethal cardiovascular diseases. It is frequently fatal and worsened by cardiac failure, shock, and malignant arrhythmia (1). Because of its acute onset, high case fatality rate and poor prognosis, MI has become one of the major challenges faced by cardiovascular doctors. Therefore, it is important to detect the mechanisms and risk factors of myocardial infarction for the prevention, diagnosis and treatment. Studies have shown that the manifestation and progression of cardiovascular diseases are linked to the intake, metabolic disorders and imbalances of trace elements (2–5).

Copper, an essential micronutrient for human health and development, is involved in various biological processes in the heart muscle that are critical to cardiac metabolism and function. In one study, dietary copper supplementation is shown to replenish cardiac copper, increase VEGF, promote angiogenesis, and reverse mouse hypertrophic cardiomyopathy (6). Furthermore, copper is crucial element of superoxide dismutase 1 (SOD1) and Cytochrome c Oxidase (CcO), which are involved in antioxidant activity and mitochondrial energy metabolism (7–9). Copper restriction in the diet leads to cardiac hypertrophy, which in turn causes heart failure (10). Capillary density decreases with the loss of Cu, and a failing heart’s myocardial mitochondria’s structure and function are altered (11, 12). In hypertrophic hearts instigated by contraction of the upper aorta and dietary Copper deficiency, Copper supplementation enhanced the contractile function as well as structural integrity (6, 13). In fundamental investigations, several studies have shown a connection between copper depletion and myocardial ischemic infarction (14). However, in clinical studies, there is insufficient evidence to prove the relationship between copper content and myocardial infarction. Previous studies investigating the correlation between copper and CVD had shown erratic results, majority of these found the correlation of serum concentrations of these elements instead of their dietary intake (15–25). In previous studies, high serum copper was correlated with cardiovascular risk (26–30). Instead of dietary intake variations, these serum levels were linked to an increase in ceruloplasmin (and serum copper) (31, 32). The relationship between copper consumption and its serum levels is complicated by the fact that copper absorption varies with age, gender, the use of oral contraceptives as well as copper intake from different food sources (20, 26). As a result, the amount of copper in the blood cannot be used to determine total body copper status or their dietary consumption (27). Nevertheless, dietary recommendations for these elements’ nutritional value cannot be influenced by the correlation between CVD and serum concentrations of these elements. As a result, dietary copper intake must be studied instead of its serum concentration to determine the risk of cardiovascular disease. However, these connections have not been addressed before. To fill the gap of this study, we collected data from the 2011 to 2018 National Health and Nutrition Examination Survey (NHANES) and conducted a comprehensive cross-sectional analysis with national representation, assessing dietary copper consumption and myocardial infarction threat to provide a theoretical basis for prevention.

## Materials and methods

### Study population

The NHANES study is a multi-stage, stratified, large, nationally representative study of the United States population. It is conducted by the Centers for Disease Control and Prevention’s National Center for Health Statistics to assess the nutritional and physical status of Americans (33). The cross-sectional survey includes data on population demographics, diet, physical examination, and questionnaires, among others. NHANES survey data is freely available on the web to be used by data researchers and other users. For more information about NHANES, visit www.cdc.gov/nchs/nhanes/.

The study involved 39,156 participants aged 20–80 years. Among these individuals, we excluded participants who did not participate in recording copper intake (*n* = 10,867), participants who did not record myocardial infarction (*n* = 11,180), participants with missing confounding data (*n* = 2,055), pregnant women (*n* = 171), and participants with large deviations in copper intake (*n* = 7). A total of 14,876 participants were included in the analysis. The NCHS Ethics Review Board approved all NHANES programs, and participants or their agents provided informed consent prior to participation (34).

### Variables

Copper intake was set as the dependent variable and MI as the independent variable. The dietary data was gathered from the NHANES database, and all participants gave two 24-h dietary recall interviews. The first recall interview was conducted at the NHANES Mobile Testing Center, and the second recall interview was conducted by telephone 3–10 days later. The daily total of all nutrients/food components was calculated using the USDA Food and Nutrition Diet Research database and entered into the NHANES database (35). In our study, we analyzed mean copper intake from two such 24-h recalls.

The incidence of MI was detected using the Health Status Questionnaire (MCQ). MI was said to have occurred when the participant answered “yes” to the question, “Has a doctor or other health professional ever told {you/SP}{you/s/he}. a heart attack (also known as a myocardial infarction (my-o-car- dee-al in-fark-shun)?” (36).

Building on previous research, we included the following relevant covariates: sex, age, education level (less than 9th grade, grades 9–11, high school graduation/GED or equivalent, some college or AA degree, college graduation or above), BMI, smoking history, drinking history, hypertension, diabetes, and lipid levels. Participants were considered to have diabetes if one of the following was true: they were informed by a physician or other health professional that they had diabetes, were taking insulin, or were taking diabetes medication to lower blood sugar (37). Participants were considered to have hypertension if they met one of the following criteria: were advised by a doctor or other healthcare professional regarding their high blood pressure or were told they were taking antihypertensive medication, and had a mean systolic blood pressure (SBP) ≥ 140 mmHg or diastolic blood pressure (DBP) ≥ 90 mmHg on examination. Detailed information on the process of obtaining dietary copper intake, MI, and other covariates can be found at www.cdc.gov/nchs/nhanes/.

### Statistical analysis

The extraction and merging of NHANES data from 2011 to 2018 was done on R Studio (version 4.1.3). Baseline characteristics of continuous variables were compared using a *t*-test or non-parametric Mann-Whitney U test, and categorical variables were compared using a chi-square test or Fisher’s test. Propensity score matching (PSM) uses 1:2 nearest neighbor matching algorithm. In 1983, Rubin and Rosenbaum proposed propensity score matching, which has been widely used in observational studies to reduce selection bias (38, 39). It is based on the concept of counterfactual and can strengthen causal arguments in observational research by reducing selection bias (40). Confounding factors such as age, sex, education level, BMI, smoking history, drinking history, hypertension, diabetes and blood lipids were selected for matching. Univariate Logistic regression was used to analyze the risk factors of myocardial infarction. Multivariate logistic regression model was used to analyze the relationship between copper intake and myocardial infarction. Possible non-linear relationships are determined by restricted cubic splines (RCS). Stratified analyses were performed according to age, sex, BMI, smoking history, hypertension, diabetes, and lipid levels. The association between copper intake and the risk of myocardial infarction was further investigated. In addition, to further test the robustness of the results, we conducted a sensitivity analysis using the inverse probability weighting of propensity scores (IPTW) method. All data analysis and graphic design were performed using R Studio (version 4.1.3), EmpowerStats,^1^ GraphPad Prism (version 8.0), and Adobe Illustrator (version 2020). *P* < 0.05 was considered statistically significant.

## Results

### Baseline characteristics of total participants according to copper intake quartiles

The average age of the subjects was 50.15 ± 17.45 years old, of whom 49.19% were male (*n* = 7,318). The median value of Copper was 1.083 mg/day. The medians of copper in quartile group were 0.65, 0.95, 1.24, and 1.79 mg/day, respectively. There were statistically significant differences in all parameters among the four groups except TC and HDL levels (Table 1).

**TABLE 1:** Baseline characteristics of total participants according to copper intake quartiles.

Characteristic	Total subjects	Copper intake quartile, mg/day				
		
		Q1 (0.0655–0.807)	Q2 (0.807–1.082)	Q3 (1.082–1.44)	Q4 (1.44–10.6205)	*P*-value
Number	14,876	3,712	3,722	3,722	3,720	
Median intake	1.083	0.65	0.95	1.24	1.79	
Age (years old)	50.15 ± 17.45	50.38 ± 18.21	50.97 ± 17.79	50.35 ± 17.23	48.90 ± 16.46	<0.001
Sex, *n* (%)						<0.001
Male	7,318 (49.19)	1,329 (35.80)	1,686 (45.30)	1,934 (51.96)	2,369 (63.68)	
Female	7,558 (50.81)	2,383 (64.20)	2,036 (54.70)	1,788 (48.04)	1,351 (36.32)	
Level of education, *n* (%)						<0.001
Less than 9th grade	1,090 (7.33)	350 (9.43)	287 (7.71)	261 (7.01)	192 (5.16)	
9–12th grade	1,758 (11.82)	604 (16.27)	469 (12.60)	366 (9.83)	319 (8.58)	
High school graduate/GED or equivalent	3,369 (22.65)	1,037 (27.94)	914 (24.56)	783 (21.04)	635 (17.07)	
Some college or AA degree	4,730 (31.80)	1,209 (32.57)	1,192 (32.03)	1,192 (32.03)	1,137 (30.56)	
College graduate or above	3,929 (26.41)	512 (13.79)	860 (23.11)	1,120 (30.09)	1,437 (38.63)	
BMI (kg/m^2^)						<0.001
<25	4,032 (27.10)	907 (24.43)	953 (25.60)	1,002 (26.92)	1,170 (31.45)	
25–30	4,775 (32.10)	1,133 (30.52)	1,164 (31.27)	1,218 (32.72)	1,260 (33.87)	
≥30	6,069 (40.80)	1,672 (45.04)	1,605 (43.12)	1,502 (40.35)	1,290 (34.68)	
Smoking history, *n* (%)						<0.001
No	8,356 (56.17)	1,937 (52.18)	2,125 (57.09)	2,149 (57.74)	2,145 (57.66)	
Yes	6,520 (43.83)	1,775 (47.82)	1,597 (42.91)	1,573 (42.26)	1,575 (42.34)	
Drinking history, *n* (%)						<0.001
No	4,945 (33.24)	1,450 (39.06)	1,281 (34.42)	1,175 (31.57)	1,039 (27.93)	
Yes	9,931 (66.76)	2,262 (60.94)	2,441 (65.58)	2,547 (68.43)	2,681 (72.07)	
Hypertension, *n* (%)						<0.001
No	8,224 (55.28)	1,911 (51.48)	1,996 (53.63)	2,083 (55.96)	2,234 (60.05)	
Yes	6,652 (44.72)	1,801 (48.52)	1,726 (46.37)	1,639 (44.04)	1,486 (39.95)	
Diabetes, *n* (%)						<0.001
No	10,671 (71.73)	2,605 (70.18)	2,590 (69.59)	2,659 (71.44)	2,817 (75.73)	
Yes	2,947 (19.81)	809 (21.79)	805 (21.63)	730 (19.61)	603 (16.21)	
IGT + IFG	1,258 (8.46)	298 (8.03)	327 (8.79)	333 (8.95)	300 (8.06)	
TC (mmol/L)	4.94 ± 1.09	4.93 ± 1.11	4.95 ± 1.11	4.95 ± 1.09	4.91 ± 1.05	0.322
TG (mmol/L)	1.72 ± 1.52	1.63 ± 1.20	1.75 ± 1.84	1.76 ± 1.56	1.74 ± 1.42	0.016
HDL (mmol/L)	1.37 ± 0.41	1.37 ± 0.43	1.37 ± 0.41	1.37 ± 0.41	1.37 ± 0.41	0.759

BMI, body mass index; TC, total cholesterol; TG, triglyceride; HDL, high density lipoprotein.

### Characteristics of participants before and after matching by myocardial infarction

A total of 655 participants had MI before matching. Except for the history of drinking, the incidence of other covariates was significantly different (*P* < 0.01). Using closest neighbor propensity score matching (1:2), we established a comparable control group to further support the relationship between dietary copper intake and MI risk(Supplementary Figures 1 and 2). There wasn’t a significant statistical difference in the majority of baseline characteristics between the two groups after propensity score matching, which matched 1,300 participants in the control group and 654 participants in the MI group (Table 2). Meanwhile, the standardized mean difference (SMD) of baseline data before and after matching in the MI group was less than 0.01, which was not statistically significant (Supplementary Table 1).

**TABLE 2:** Characteristics of participants before and after matching by myocardial infarction.

Variables	Before matching			After matching			
		
	Control group (*n* = 14,221)	MI group (*n* = 655)	*P*-value	Control group (*n* = 1,300)	MI group (*n* = 654)	SMD	*P*-value
Age (years old)	49.38 ± 17.29	66.89 ± 11.36	<0.001	64.79 ± 14.05	66.87 ± 11.36	0.16 (0.07, 0.26)	0.043
Sex, *n* (%)			<0.001			0.06(-0.03, 0.16)	0.186
Male	6,892 (48.46)	426 (65.04)		805 (61.92)	425 (64.98)		
Female	7,329 (51.54)	229 (34.96)		495 (38.08)	229 (35.02)		
Level of education, *n* (%)			<0.001			0.10 (0.01, 0.20)	0.332
Less than 9th grade	1,011 (7.11)	79 (12.06)		151 (11.62)	78 (11.93)		
9–12th grade	1,660 (11.67)	98 (14.96)		205 (15.77)	98 (14.98)		
High school graduate/GED or equivalent	3,185 (22.40)	184 (28.09)		326 (25.08)	184 (28.13)		
Some college or AA degree	4,543 (31.95)	187 (28.55)		361 (27.77)	187 (28.59)		
College graduate or above	3,822 (26.88)	107 (16.34)		257 (19.77)	107 (16.36)		
BMI (kg/m^2^)			<0.001			0.07(-0.02, 0.17)	0.328
<25	3,898 (27.41)	134 (20.46)		243 (18.69)	134 (20.49)		
25–30	4,569 (32.13)	206 (31.45)		451 (34.69)	206 (31.50)		
≥30	5,754 (40.46)	315 (48.09)		606 (46.62)	314 (48.01)		
Smoking history, *n* (%)			<0.001			0.10 (0.00, 0.19)	0.044
No	8,133 (57.19)	223 (34.05)		504 (38.77)	223 (34.10)		
Yes	6,088 (42.81)	432 (65.95)		796 (61.23)	431 (65.90)		
Drinking history, *n* (%)			0.537			0.05(-0.04, 0.15)	0.259
No	4,720 (33.19)	225 (34.35)		479 (36.85)	224 (34.25)		
Yes	9,501 (66.81)	430 (65.65)		821 (63.15)	430 (65.75)		
Hypertension, *n* (%)			<0.001			0.07(-0.02, 0.17)	0.128
No	8,101 (56.97)	123 (18.78)		283 (21.77)	123 (18.81)		
Yes	6,120 (43.03)	532 (81.22)		1,017 (78.23)	531 (81.19)		
Diabetes, *n* (%)			<0.001			0.19 (0.09, 0.28)	<0.001
No	10,372 (72.93)	299 (45.65)		671 (51.62)	299 (45.72)		
Yes	2,643 (18.59)	304 (46.41)		490 (37.69)	304 (46.48)		
IGT + IFG	1,206 (8.48)	52 (7.94)		139 (10.69)	51 (7.80)		
TC (mmol/L)	4.96 ± 1.08	4.44 ± 1.15	<0.001	4.58 ± 1.06	4.44 ± 1.14	0.12 (0.03, 0.22)	0.010
TG (mmol/L)	1.71 ± 1.53	1.89 ± 1.28	0.004	1.86 ± 1.48	1.89 ± 1.28	0.02(-0.07, 0.12)	0.637
HDL (mmol/L)	1.38 ± 0.41	1.27 ± 0.41	<0.001	1.29 ± 0.39	1.27 ± 0.41	0.06(-0.04, 0.15)	0.233
Copper intake quartiles (%)			<0.001			0.13 (0.04, 0.23)	0.054
Q1 (0.0655–0.807)	3,506 (24.65)	206 (31.45)		303 (23.31)	185 (28.29)		
Q2 (0.807–1.082)	3,555 (25.00)	167 (25.50)		332 (25.54)	157 (24.01)		
Q3 (1.082–1.44)	3,564 (25.06)	158 (24.12)		322 (24.77)	166 (25.38)		
Q4 (1.44–10.6205)	3,596 (25.29)	124 (18.93)		343 (26.38)	146 (22.32)		

### Effects of various factors on myocardial infarction by univariate analysis

**FIGURE 1:**
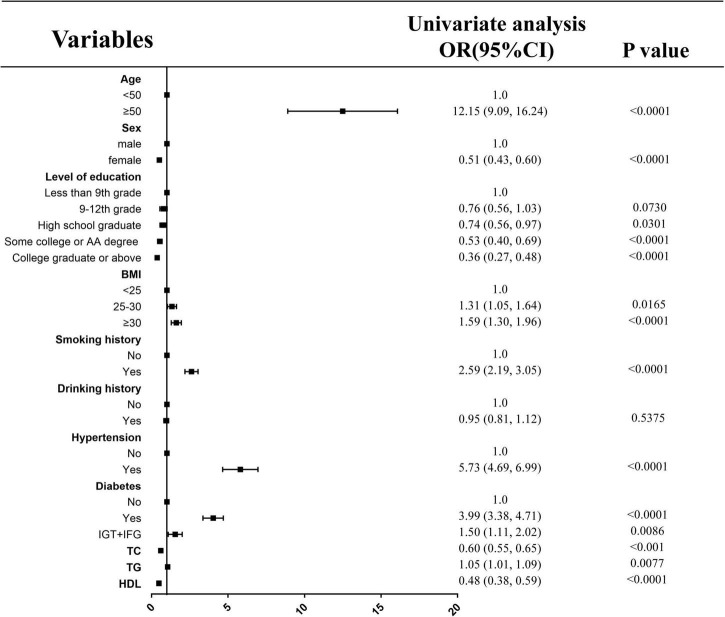
Effects of various factors on myocardial infarction by univariate analysis.

With the exception of drinking history, all other parameters were correlated with myocardial infarction. The risk of myocardial infarction (95% confidence interval) among participants aged ≥ 50 years was 12.15 (9.09, 16.24) compared with participants younger than 50 years. The odds ratio (95% confidence interval) for this correlation was 0.51 (0.43, 0.60) for female relative to male. The incidence of MI was 1.31 (1.05, 1.64) and 1.59 (1.30, 1.96) for 25 ≤ BMI < 30 Kg/m^2^ and ≥ 30 Kg/m^2^ compared with BMI < 25 Kg/m^2^, respectively, as depicted in Figure 1 as well as in Table 3.

**TABLE 3:** Effects of various factors on myocardial infarction by univariate analysis.

Variables	Statistics	OR (95%CI)	*P*-value
**Age (years old)**			
<50	7,175(48.23)	1.0	
≥50	7,701 (51.77)	12.15 (9.09, 16.24)	<0.0001
**Sex, *n* (%)**			
Male	7,318 (49.19)	1.0	
Female	7,558 (50.81)	0.51 (0.43, 0.60)	<0.0001
**Level of education, *n* (%)**			
Less than 9th grade	1,090 (7.33)	1.0	
9–12th grade	1,758 (11.82)	0.76 (0.56, 1.03)	0.0730
High school graduate/GED or equivalent	3,369 (22.65)	0.74 (0.56, 0.97)	0.0301
Some college or AA degree	4,730 (31.80)	0.53 (0.40, 0.69)	<0.0001
College graduate or above	3,929 (26.41)	0.36 (0.27, 0.48)	<0.0001
**BMI (kg/m^2^)**			
<25	4,032 (27.10)	1.0	
25–30	4,775 (32.10)	1.31 (1.05, 1.64)	0.0165
≥30	6,069 (40.80)	1.59 (1.30, 1.96)	<0.0001
**Smoking history, *n* (%)**			
No	8,356 (56.17)	1.0	
Yes	6,520 (43.83)	2.59 (2.19, 3.05)	<0.0001
**Drinking history, *n* (%)**			
No	4,945 (33.24)	1.0	
Yes	9,931 (66.76)	0.95 (0.81, 1.12)	0.5375
**Hypertension, *n* (%)**			
No	8,224 (55.28)	1.0	
Yes	6,652 (44.72)	5.73 (4.69, 6.99)	<0.0001
**Diabetes, *n* (%)**			
No	10,671 (71.73)	1.0	
Yes	2,947 (19.81)	3.99 (3.38, 4.71)	<0.0001
IGT + IFG	1,258 (8.46)	1.50 (1.11, 2.02)	0.0086
TC (mmol/L)	4.94 ± 1.09	0.60 (0.55, 0.65)	<0.001
TG (mmol/L)	1.72 ± 1.52	1.05 (1.01, 1.09)	0.0077
HDL (mmol/L)	1.37 ± 0.41	0.48 (0.38, 0.59)	<0.0001

### Correlation analysis of copper intake and myocardial infarction before and after matching

Before matching, without adjusting for any confounders, the odds ratios for the correlation of copper with myocardial infarction compared with Q1 were 0.80 (0.65, 0.99), 0.75 (0.61, 0.93), and 0.59 (0.47, 0.74) in univariate logistic regression, respectively. After adjusting for confounders (age, sex, level of education, BMI, smoking history, hypertension, diabetes, TC, TG and HDL), the differences in the correlation with MI among the other three groups were 0.78 (0.62, 0.97), 0.78 (0.62, 0.98), 0.69 (0.53, 0.88), respectively (Table 4).

**TABLE 4:** Correlation analysis between copper intake and myocardial infarction before matching.

	Model 1 β (95% CI) *p* value	Model 2 β (95% CI) *p* value	Model 3 β (95% CI) *p* value
Copper	0.70 (0.60, 0.82) <0.0001	0.76 (0.64, 0.89) 0.0007	0.78 (0.67, 0.92) 0.0023
**Copper (mg/d) quartiles**			
Q1 (0.0655–0.807)	1.0	1.0	1.0
Q2 (0.807–1.082)	0.80 (0.65, 0.99) 0.0362	0.76 (0.61, 0.94) 0.0127	0.78 (0.62, 0.97) 0.0284
Q3 (1.082–1.44)	0.75 (0.61, 0.93) 0.0094	0.75 (0.60, 0.93) 0.0109	0.78 (0.62, 0.98) 0.0366
Q4 (1.44–10.6205)	0.59 (0.47, 0.74) <0.0001	0.65 (0.51, 0.83) 0.0005	0.69 (0.53, 0.88) 0.0030

Model 1: No adjustments made for confounding factors.

Model 2: Adjustments made for age, sex, level of education and BMI.

Model 3: Adjustments same as that in model 2 plus hypertension, diabetes, smoking history, TC, TG, and HDL.

After matching without adjusting for any confounders, in univariate logistic regression, the odds ratio for the correlation of copper with myocardial infarction was 0.70 (0.53, 0.91) in group Q4 compared with group Q1, and the remaining two groups were not statistically different. After adjusting for confounders (age, sex, level of education, BMI, smoking history, hypertension, diabetes, TC, TG and HDL), the odds ratio for the correlation between copper and myocardial infarction in the Q4 group was 0.70 (0.53, 0.93), and this correlation was not significant in the other groups (Table 5).

**TABLE 5:** Correlation analysis between copper intake and myocardial infarction after matching.

	Model 1 β (95% CI) *p* value	Model 2 β (95% CI) *p* value	Model 3 β (95% CI) *p* value
Copper	0.79 (0.67, 0.93) 0.0046	0.79 (0.67, 0.94) 0.0064	0.79 (0.67, 0.94) 0.0063
**Copper (mg/d) quartiles**			
Q1 (0.0655–0.807)	1.0	1.0	1.0
Q2 (0.807–1.082)	0.77 (0.60, 1.01) 0.0574	0.77 (0.59, 1.00) 0.0528	0.77 (0.59, 1.01) 0.0554
Q3 (1.082–1.44)	0.84 (0.65, 1.10) 0.2052	0.82 (0.63, 1.08) 0.1554	0.83 (0.63, 1.08) 0.1628
Q4 (1.44–10.6205)	0.70 (0.53, 0.91) 0.0079	0.70 (0.53, 0.93) 0.0124	0.70 (0.53, 0.93) 0.0125

Model 1: No adjustments made for confounding factors.

Model 2: Adjustments made for age, sex, level of education and BMI.

Model 3: Adjustments same as that in model 2 plus hypertension, diabetes, smoking history, TC, TG, and HDL.

**FIGURE 2:**
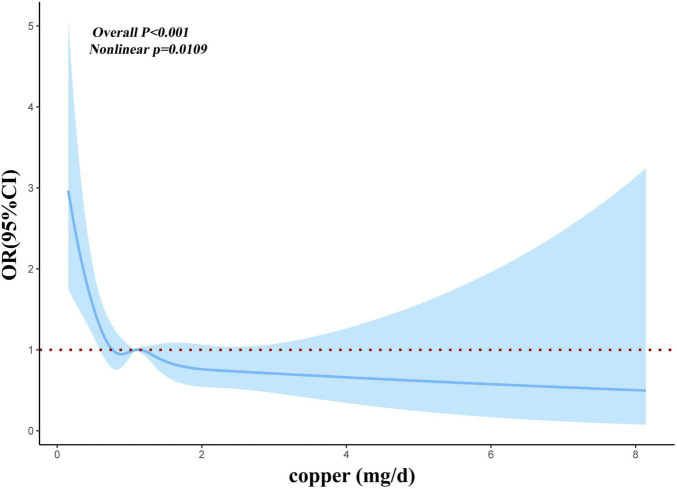
Restricted cubic spline models for the relationship between dietary copper intake and the risk of myocardial infarction before matching.

**FIGURE 3:**
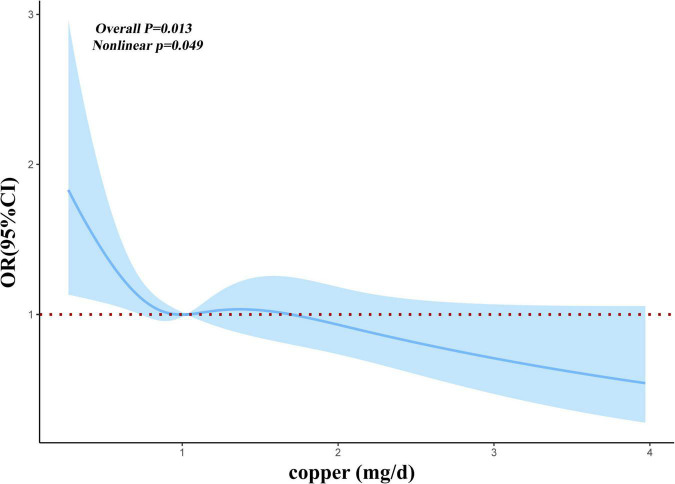
Restricted cubic spline models for the relationship between dietary copper intake and the risk of myocardial infarction after matching.

Before and after matching, we conducted RCS analysis to better understand the correlation between the risk of myocardial infarction and dietary copper intake. Before matching, we discovered an L-shaped connection between dietary copper consumption and risk of myocardial infarction in adjusted models (Figure 2). With increased dietary copper intake, there was a non-linear decrease in the prevalence of myocardial infarction (*P* for non-linear = 0.0109). Results from the RCS model (Figure 3) similarly revealed a non-linear association between dietary copper intake and the threat of myocardial infarction after matching (*P* for non-linear = 0.049).

### Stratified analysis before and after matching

**FIGURE 4:**
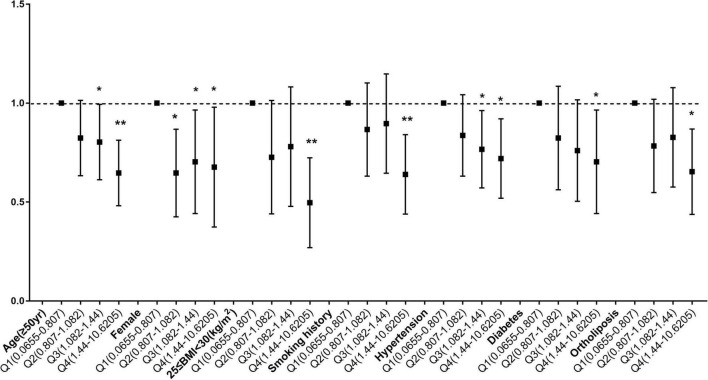
Subgroup analysis of dose-related copper intake and myocardial infarction before matching. *p < 0.05, **p < 0.01.

**FIGURE 5:**
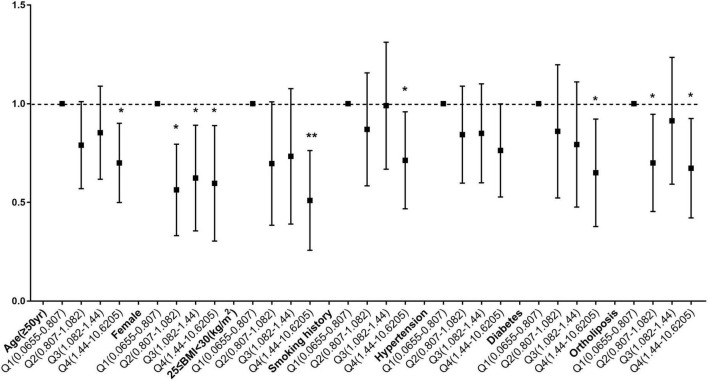
Subgroup analysis of dose-related copper intake and myocardial infarction after matching. *p < 0.05, **p < 0.01.

To determine whether the relationship between copper intake and myocardial infarction varied by age, sex, BMI, smoking status, hypertension, diabetes, and lipid profiles, stratified analyses were employed. When age was used in stratified analysis, the correlation between copper intake and myocardial infarction was more substantial in those aged ≥ 50 years, with odds ratios of 0.74 (0.62, 0.88) and 0.77 (0.65, 0.92) before and after matching, respectively. There were more significant for the correlation of copper with myocardial infarction Q4 compared with Q1, with odds ratios of 0.63 (0.49, 0.82) and 0.68 (0.51, 0.91) before and after matching (Supplementary Table 2). Likewise, female (Supplementary Table 3), 25 ≤ BMI < 30 (Supplementary Table 4), smokers (Supplementary Table 5), hypertensive patients (Supplementary Table 6), diabetic patients (Supplementary Table 7) and People with normal blood lipids (Supplementary Table 8) had a stronger correlation between copper consumption and myocardial infarction. Before matching (Figure 4) and after matching (Figure 5), the correlation of copper intake in group Q4, with a dose-response relationship, was more significant than that in group Q1.

### Sensitivity analysis

The baseline data of the two groups were weighted by inverse probability of treatment weighting (IPTW) using the propensity score for possible confounding factors, such as age, sex, level of education, BMI, smoking history, drinking history, hypertension, diabetes and blood lipids. After weighting, the data of the two groups were well balanced and comparable except for age (Supplementary Table 9). Myocardial infarction was used as the dependent variable and weighted copper intake was used as the independent variable in a multivariate logistic regression analysis. After adjusting for confounders, there was a significant negative correlation between the two groups, indicating that the above results were robust (Supplementary Table 10).

## Discussion

The results of this study preliminarily verified the new evolutionary biological view of myocardial infarction: copper could prevent myocardial infarction (41). A few previous studies had reported the relationship between dietary copper intake and myocardial infarction, which was consistent with the results of this study. Hanish et al. (1) used Mendelian randomization to assess that copper could reduce the risk of IHD, which was consistent with the suggestion more than 40 years ago. Although this hypothesis has long existed, it has been largely ignored, and to our knowledge no large studies have evaluated the effect of copper on myocardial infarction.

Here, we analytically reviewed large population data of 14,876 cases between 2011 and 2018 to explore the probable correlation between copper consumption and the risk of myocardial infarction. The study had some major findings. First and in line with earlier studies, we found that for myocardial infarction: age, male gender, overweight and obesity, smoking, hypertension, diabetes and blood lipids level were risk factors. The present study’s most significant conclusion was that the risk of myocardial infarction decreased with an increase of dietary copper intake before and after matching. It was proposed that copper intake might be a preventative measure against myocardial infarction when combined with the findings of RCS analysis. Additionally, stratified analysis further revealed that dietary copper intake was a important protective characteristic for people aged ≥ 50 years, female, 25 ≤ BMI < 30, with history of smoking, hypertension, diabetes and ortholiposis.

Copper, as a powerful antioxidant, might act as preventive measure in the progress of atherosclerosis which underlined cardiovascular diseases. According to Klevay et al. (42), copper was an antioxidant, a constituent of monoamine oxidase and SOD, which benefited the blood vessel wall’s fiber’s by maintaining their toughness and suppleness. Copper had been shown to promote cardiac regeneration by reactivating hypoxia inducible factor 1-regulated angiogenesis, constituting another therapeutic strategy for ischemic heart disease (43). Molecular biology studies had shown that copper functioned in different organelles as a cofactor for proteins and enzymes required for cytoplasmic maturation and enzyme production (44). Copper deficiency leaded to cholesterol breakdown and disturbance of plasma lipoprotein metabolism, resulting in cholesterol deposition in damaged blood vessels and atherosclerosis. Here, we found an inverse correlation between dietary copper intake and the threat of myocardial infarction, further emphasizing the protective effect of antioxidant minerals on cardiovascular diseases. Our findings were consistent with those of Kodali et al. (1): Copper might reduce the risk of ischemic heart disease. Yin et al. (45) showed that high levels of antioxidant micronutrients in the diet were correlated with reduced cardiovascular disease morbidity; levels of iron, zinc, and copper were inversely and non-linearly correlated with cardiovascular diseases. Therefore, trace minerals might have the ability to prevent cardiovascular disease.

This study also made a lot of subgroup analysis, which had explored the impact of copper intake on different populations. It was found that copper intake had a significant impact on myocardial infarction in elderly people (whose age ≥ 50 years old). This might be due to the increased risk of micronutrient deficiency in the elderly caused by pathophysiological changes (46). Although the plasma copper level was in the normal range, the availability of intracellular copper ions was reduced, thus older individuals were more prone to a range of cardiovascular diseases when they were copper deficient. Therefore, dietary copper element supplementation was appropriate for the elderly (47). By gender subgroup analysis, the effect of dietary copper intake on myocardial infarction was found to be slightly more significant in women than in men (0.80 > 0.73), possibly because the effect of Cu absorption was consistently 10% higher in women than in men (27). As one of the important risk factors of myocardial infarction, hypertension was also closely related to copper ions. This study found that copper intake was more significant in hypertensive population with myocardial infarction, and there had been numerous studies on the mechanisms of copper and the development of hypertension in a series of ways, since copper was required in several enzymatic functions to maintain the integrity of the vascular system (48). A crucial enzyme in the control of blood pressure, angiotensin-converting enzyme (ACE), could be inhibited by copper at the same time (49). Copper deficiency was also correlated with impaired endothelium-dependent arterial relaxation which could lead to hypertension, possibly due to reduced activity of copper zinc superoxide dismutase (21). In addition, copper participated in the synthesis of dehydroepiandrosterone (DHEA) through oxidation of cholesterol. Therefore, the decrease of DHEA level due to copper deficiency might lead to the development of hypertension (50). Diabetes was one of the risk factors for myocardial infarction, and there were a large number of studies on Cu ions and diabetes which were consistent with the results of this study. Increased amounts of triglyceride and cholesterol production, insulin, and peroxidation damage, release may result from Cu deficiency when combined with glucose, fructose, or iron ingestion (51). Different studies had been directly or indirectly targeting Cu ions to reverse diabetic complications, and there were also results showing that classical hypoglycemic drugs had the ability to chelate Cu ions. In different cell lines, smoking could cause direct or indirect oxidative stress and copper, as a consistent antioxidant, could prevent some health issues instigated by smoking, such as lung disease, kidney failure and diabetes (52). The meta-analysis results of Wang et al. (53) were consistent with the subgroup analysis of blood lipid levels, and dietary copper had no effect on people with dyslipidemia, which may be related to the regulatory mechanism of Cu in the body.

This study also has some limitations. First of all, because it is a cross-sectional study, causal inference is not possible. Further prospective studies are required to confirm these findings. Secondly, the inclusion criteria of myocardial infarction depend on the self-reported history of myocardial infarction, and their impact on the sub-types and stages of myocardial infarction was still unclear. Despite the fact that we have incorporated numerous covariates into our analytic model, we are unable to completely rule out the influence of unidentified confounding factors. Therefore, to elucidate the correlation between dietary copper intake and risk of MI, larger clinical studies on different stages and sub-types of MI are required in the future.

## Conclusion

This is a large cross-sectional study based on the NHANES database to estimate the association between dietary copper intake and the risk of MI. We found that the risk of MI decreased with increasing dietary copper intake. In addition, copper intake was found to be more protective in elderly-aged women, overweight individuals, hypertensives, smokers, and diabetic patients. It is speculated that MI may be prevented by adjusting dietary copper intake, the protective action of which, has a theoretical basis.

## Data availability statement

The datasets presented in this study can be found in online repositories. The names of the repository/repositories and accession number(s) can be found below: National Health and Nutrition Examination Survey (NHANES).

## Ethics statement

The NHANES study was approved by the Research Ethics Review Board of the National Center for Health Statistics, and all participants signed informed consent forms. Access to the NHANES database does not require any ethical or administrative rights. More details are available online, at www.cdc.gov/nchs/nhanes/. The patients/participants provided their written informed consent to participate in this study. Written informed consent was obtained from the individual(s) for the publication of any potentially identifiable images or data included in this article.

## Author contributions

HW and XN proposed the strategy and implemented the extraction and collation of the data. XN, NS, and RZ validated and analyzed the data. HW and LH drafted the manuscript. QW and YL revised and finalized the manuscript. All authors approved the final version of the manuscript.

## Footnotes

1
http://www.empowerstats.com
