# Supplementary material for: Dietary copper intake and risk of myocardial infarction in US adults: A propensity score-matched analysis
Source: Front Cardiovasc Med. 2022 Nov 10;9:942000. doi: 10.3389/fcvm.2022.942000 (PMC9685336; doi:10.3389/fcvm.2022.942000)
Supplement: Supplementary file 1 [file Table_1.DOC]

### **Table S1 Baseline characteristics of myocardial infarction group before and after matching**

| **Variables** | **Before matching**  **(MI Group)** | **After matching**  **(MI Group)** | **SMD** | **P-value** |
| --- | --- | --- | --- | --- |
| **Age(years old)** | 66.89±11.36 | 66.87±11.36 | 0.00 (-0.11, 0.11) | 0.976 |
| **Sex, n(%)** |  |  | 0.00 (-0.11, 0.11) | 0.984 |
| Male | 426 (65.04) | 425 (64.98) |  |  |
| Female | 229 (34.96) | 229 (35.02) |  |  |
| **Level of education, n(%)** |  |  | 0.00 (-0.11, 0.11) | 1.000 |
| Less than 9th grade | 79 (12.06) | 78 (11.93) |  |  |
| 9-12th grade | 98 (14.96) | 98 (14.98) |  |  |
| High school graduate/GED or equivalent | 184 (28.09) | 184 (28.13) |  |  |
| Some college or AA degree | 187 (28.55) | 187 (28.59) |  |  |
| College graduate or above | 107 (16.34) | 107 (16.36) |  |  |
| **BMI(kg/m^2^)** |  |  | 0.00 (-0.11, 0.11) | 1.000 |
| ＜25 | 134 (20.46) | 134 (20.49) |  |  |
| 25-30 | 206 (31.45) | 206 (31.50) |  |  |
| ≥30 | 315 (48.09) | 314 (48.01) |  |  |
| **Smoking history, n(%)** |  |  | 0.00 (-0.11, 0.11) | 0.984 |
| No | 223 (34.05) | 223 (34.10) |  |  |
| Yes | 432 (65.95) | 431 (65.90) |  |  |
| **Drinking history, n(%)** |  |  | 0.00 (-0.11, 0.11) | 0.969 |
| No | 225 (34.35) | 224 (34.25) |  |  |
| Yes | 430 (65.65) | 430 (65.75) |  |  |
| **Hypertension, n(%)** |  |  | 0.00 (-0.11, 0.11) | 0.989 |
| No | 123 (18.78) | 123 (18.81) |  |  |
| Yes | 532 (81.22) | 531 (81.19) |  |  |
| [**Diabetes**](javascript:;)**, n(%)** |  |  | 0.01 (-0.10, 0.11) | 0.996 |
| No | 299 (45.65) | 299 (45.72) |  |  |
| Yes | 304 (46.41) | 304 (46.48) |  |  |
| IGT+IFG | 52 (7.94) | 51 (7.80) |  |  |
| **TC(mmol/L)** | 4.44 ± 1.15 | 4.44±1.14 | 0.00 (-0.11, 0.11) | 0.967 |
| **TG(mmol/L)** | 1.89 ± 1.28 | 1.89±1.28 | 0.00 (-0.11, 0.11) | 0.981 |
| **HDL(mmol/L)** | 1.27 ± 0.41 | 1.27±0.41 | 0.00 (-0.11, 0.11) | 0.991 |
| **Copper intake quartiles(%)** |  |  | 0.00 (-0.11, 0.11) | 0.984 |
| Q1（0.0655-0.807） | 206 (31.45) | 185 (28.29) |  |  |
| Q2（0.807-1.082） | 167 (25.50) | 157 (24.01) |  |  |
| Q3（1.082-1.44） | 158 (24.12) | 166 (25.38) |  |  |
| Q4（1.44-10.6205） | 124 (18.93) | 146 (22.32) |  |  |
