# Supplementary material for: Dietary copper intake and risk of myocardial infarction in US adults: A propensity score-matched analysis
Source: Front Cardiovasc Med. 2022 Nov 10;9:942000. doi: 10.3389/fcvm.2022.942000 (PMC9685336; doi:10.3389/fcvm.2022.942000)
Supplement: Supplementary file 2 [file Table_2.DOC]

### **Table S2 Association between copper intake and myocardial infarction as categorized by age**

| **Subgroup** | **Before Matching** | | **After Matching** | |
| --- | --- | --- | --- | --- |
|  | **OR(95%CI)** | **P-value** | **OR(95%CI)** | **P-value** |
| **age(years old)** |  |  |  |  |
| <50 | 0.83 (0.49, 1.41) | 0.4893 | 0.82 (0.41, 1.61) | 0.5573 |
| Q1 | 1.0 |  | 1.0 |  |
| Q2 | 0.55 (0.24, 1.25) | 0.1552 | 1.15 (0.41, 3.25) | 0.7853 |
| Q3 | 0.60 (0.27, 1.34) | 0.2095 | 0.99 (0.35, 2.83) | 0.9913 |
| Q4 | 0.75 (0.34, 1.63) | 0.4643 | 0.75 (0.28, 2.03) | 0.5754 |
| **≥50** | **0.74 (0.62, 0.88)** | **0.0005** | **0.77 (0.65, 0.92)** | **0.0047** |
| Q1 | 1.0 |  | 1.0 |  |
| Q2 | 0.81 (0.64, 1.02) | 0.0755 | 0.77 (0.58, 1.02) | 0.0669 |
| Q3 | **0.79 (0.62, 1.00)** | **0.0483** | 0.83 (0.63, 1.10) | 0.1999 |
| Q4 | **0.63 (0.49, 0.82)** | **0.0005** | **0.68 (0.51, 0.91)** | **0.0108** |

Multivariable model is adjusted for sex, level of education, BMI, hypertension, diabetes, smoking history, TC, TG and HDL
