# Supplementary material for: Dietary copper intake and risk of myocardial infarction in US adults: A propensity score-matched analysis
Source: Front Cardiovasc Med. 2022 Nov 10;9:942000. doi: 10.3389/fcvm.2022.942000 (PMC9685336; doi:10.3389/fcvm.2022.942000)
Supplement: Supplementary file 3 [file Table_3.DOC]

### **Table S3 Association between copper intake and myocardial infarction as categorized by sex**

| **Subgroup** | **Before Matching** | | **After Matching** | |
| --- | --- | --- | --- | --- |
|  | **OR(95%CI)** | **P-value** | **OR(95%CI)** | **P-value** |
| **Sex** |  |  |  |  |
| Male | 0.80 (0.67, 0.96) | 0.0172 | 0.82 (0.67, 0.99) | 0.0433 |
| Q1 | 1.0 |  | 1.0 |  |
| Q2 | 0.93 (0.69, 1.26) | 0.6560 | 1.00 (0.70, 1.42) | 0.9783 |
| Q3 | 0.89 (0.66, 1.20) | 0.4492 | 1.05 (0.74, 1.49) | 0.7923 |
| Q4 | 0.75 (0.55, 1.02) | 0.0694 | 0.82 (0.58, 1.17) | 0.2767 |
| **Female** | **0.73 (0.53, 1.00)** | **0.0501** | **0.69 (0.48, 1.00)** | **0.0491** |
| Q1 | 1.0 |  | 1.0 |  |
| Q2 | **0.62 (0.44, 0.88)** | **0.0072** | **0.53 (0.35, 0.81)** | **0.0030** |
| Q3 | **0.67 (0.46, 0.98)** | **0.0381** | **0.58 (0.38, 0.91)** | **0.0171** |
| Q4 | **0.63 (0.40, 1.00)** | **0.0488** | **0.55 (0.33, 0.91)** | **0.0205** |

Multivariable model is adjusted for age, level of education, BMI, smoking history, hypertension, diabetes, TC, TG and HDL
