# Supplementary material for: Dietary copper intake and risk of myocardial infarction in US adults: A propensity score-matched analysis
Source: Front Cardiovasc Med. 2022 Nov 10;9:942000. doi: 10.3389/fcvm.2022.942000 (PMC9685336; doi:10.3389/fcvm.2022.942000)
Supplement: Supplementary file 4 [file Table_4.DOC]

### **Table S4 Association between copper intake and myocardial infarction as categorized by BMI**

| **Subgroup** | **Before Matching** | | **After Matching** | |
| --- | --- | --- | --- | --- |
|  | **OR(95%CI)** | **P-value** | **OR(95%CI)** | **P-value** |
| **BMI (kg/m^2^)** |  |  |  |  |
| ＜25 | 0.84 (0.61, 1.17) | 0.3057 | 0.93 (0.64, 1.33) | 0.6772 |
| Q1 | 1.0 |  | 1.0 |  |
| Q2 | 0.83 (0.51, 1.35) | 0.4456 | 0.70 (0.37, 1.32) | 0.2740 |
| Q3 | 0.53 (0.31, 0.92) | 0.0247 | 0.72 (0.39, 1.35) | 0.3126 |
| Q4 | 0.64 (0.38, 1.09) | 0.0981 | 0.64 (0.34, 1.23) | 0.1837 |
| 25-30 | **0.58 (0.42, 0.81)** | **0.0013** | **0.55 (0.38, 0.80)** | **0.0017** |
| Q1 | 1.0 |  | 1.0 |  |
| Q2 | 0.69 (0.46, 1.03) | 0.0705 | 0.65 (0.41, 1.03) | 0.0676 |
| Q3 | 0.74 (0.50, 1.10) | 0.1397 | 0.68 (0.42, 1.10) | 0.1194 |
| Q4 | **0.46 (0.29, 0.74)** | **0.0013** | **0.47 (0.28, 0.78)** | **0.0037** |
| ≥30 | 0.88 (0.71, 1.08) | 0.2162 | 0.88 (0.70, 1.11) | 0.2831 |
| Q1 | 1.0 |  | 1.0 |  |
| Q2 | 0.84 (0.60, 1.16) | 0.2862 | 0.91 (0.61, 1.34) | 0.6229 |
| Q3 | 0.93 (0.67, 1.30) | 0.6665 | 0.97 (0.66, 1.44) | 0.8944 |
| Q4 | 0.90 (0.63, 1.29) | 0.5826 | 0.96 (0.64, 1.44) | 0.8539 |

Multivariable model is adjusted for age, sex, level of education, smoking history, hypertension, diabetes, TC, TG and HDL
