# Supplementary material for: Dietary copper intake and risk of myocardial infarction in US adults: A propensity score-matched analysis
Source: Front Cardiovasc Med. 2022 Nov 10;9:942000. doi: 10.3389/fcvm.2022.942000 (PMC9685336; doi:10.3389/fcvm.2022.942000)
Supplement: Supplementary file 5 [file Table_5.DOC]

### **Table S5 Association between copper intake and myocardial infarction as categorized by smoking history**

| **Subgroup** | **Before Matching** | | **After Matching** | |
| --- | --- | --- | --- | --- |
|  | **OR(95%CI)** | **P-value** | **OR(95%CI)** | **P-value** |
| **Smoking history** |  |  |  |  |
| No | 0.80 (0.61, 1.04) | 0.0979 | 0.80 (0.60, 1.08) | 0.1435 |
| Q1 | 1.0 |  | 1.0 |  |
| Q2 | 0.68 (0.47, 1.00) | 0.0520 | 0.68 (0.43, 1.07) | 0.0952 |
| Q3 | 0.64 (0.43, 0.96) | 0.0324 | 0.64 (0.40, 1.04) | 0.0694 |
| Q4 | 0.80 (0.53, 1.21) | 0.2968 | 0.71 (0.44, 1.14) | 0.1529 |
| **Yes** | **0.77 (0.63, 0.93)** | **0.0080** | **0.78 (0.64, 0.96)** | **0.0208** |
| Q1 | 1.0 |  | 1.0 |  |
| Q2 | 0.85 (0.64, 1.11) | 0.2354 | 0.84 (0.60, 1.17) | 0.3019 |
| Q3 | 0.87 (0.66, 1.16) | 0.3452 | 0.95 (0.69, 1.33) | 0.7780 |
| Q4 | **0.62 (0.45, 0.85)** | **0.0027** | **0.69 (0.48, 0.97)** | **0.0352** |

Multivariable model is adjusted for age, sex, level of education, BMI, hypertension, diabetes, TC, TG and HDL
