# Supplementary material for: Dietary copper intake and risk of myocardial infarction in US adults: A propensity score-matched analysis
Source: Front Cardiovasc Med. 2022 Nov 10;9:942000. doi: 10.3389/fcvm.2022.942000 (PMC9685336; doi:10.3389/fcvm.2022.942000)
Supplement: Supplementary file 6 [file Table_6.DOC]

### **Table S6 Association between copper intake and myocardial infarction as categorized by Hypertension**

| **Subgroup** | **Before Matching** | | **After Matching** | |
| --- | --- | --- | --- | --- |
|  | **OR(95%CI)** | **P-value** | **OR(95%CI)** | **P-value** |
| **Hypertension** |  |  |  |  |
| No | 0.87 (0.62, 1.23) | 0.4421 | 0.74 (0.49, 1.13) | 0.1641 |
| Q1 | 1.0 |  | 1.0 |  |
| Q2 | 0.63 (0.36, 1.09) | 0.0993 | 0.57 (0.28, 1.16) | 0.1227 |
| Q3 | 0.90 (0.54, 1.50) | 0.6896 | 0.79 (0.41, 1.54) | 0.4948 |
| Q4 | 0.64 (0.37, 1.13) | 0.1260 | 0.48 (0.24, 0.96) | 0.0376 |
| **Yes** | **0.76 (0.64, 0.91)** | **0.0031** | **0.79 (0.65, 0.95)** | **0.0131** |
| Q1 | 1.0 |  | 1.0 |  |
| Q2 | 0.82 (0.64, 1.05) | 0.1118 | 0.82 (0.61, 1.10) | 0.1840 |
| Q3 | **0.75 (0.58, 0.97)** | **0.0271** | 0.83 (0.61, 1.11) | 0.2084 |
| Q4 | **0.70 (0.53, 0.93)** | **0.0126** | 0.74 (0.54, 1.01) | 0.0587 |

Multivariable model is adjusted for age, sex, level of education, BMI, smoking history, diabetes, TC, TG and HDL
