# Supplementary material for: Dietary copper intake and risk of myocardial infarction in US adults: A propensity score-matched analysis
Source: Front Cardiovasc Med. 2022 Nov 10;9:942000. doi: 10.3389/fcvm.2022.942000 (PMC9685336; doi:10.3389/fcvm.2022.942000)
Supplement: Supplementary file 7 [file Table_7.DOC]

### **Table S7 Association between copper intake and myocardial infarction as categorized by diabetes**

| **Subgroup** | **Before Matching** | | **After Matching** | |
| --- | --- | --- | --- | --- |
|  | **OR(95%CI)** | **P-value** | **OR(95%CI)** | **P-value** |
| **Diabetes** |  |  |  |  |
| No | 0.79 (0.63, 1.00) | 0.0491 | 0.81 (0.63, 1.04) | 0.1001 |
| Q1 | 1.0 |  | 1.0 |  |
| Q2 | 0.75 (0.53, 1.05) | 0.0921 | 0.68 (0.46, 1.02) | 0.0624 |
| Q3 | 0.83 (0.60, 1.16) | 0.2863 | 0.82 (0.55, 1.23) | 0.3383 |
| Q4 | 0.69 (0.49, 0.99) | 0.0436 | 0.71 (0.47, 1.06) | 0.0932 |
| **Yes** | **0.75 (0.59, 0.96)** | **0.0203** | **0.73 (0.55, 0.95)** | **0.0189** |
| Q1 | 1.0 |  | 1.0 |  |
| Q2 | 0.79 (0.58, 1.10) | 0.1627 | 0.81 (0.55, 1.22) | 0.3164 |
| Q3 | 0.73 (0.52, 1.03) | 0.0694 | 0.75 (0.50, 1.13) | 0.1735 |
| **Q4** | **0.67 (0.46, 0.98)** | **0.0391** | **0.61 (0.40, 0.94)** | **0.0267** |
| IGT+IFG | 0.85 (0.52, 1.39) | 0.5200 | 0.91 (0.57, 1.44) | 0.6802 |
| Q1 | 1.0 |  | 1.0 |  |
| Q2 | 0.78 (0.34, 1.75) | 0.5416 | 0.89 (0.33, 2.39) | 0.8207 |
| Q3 | 0.85 (0.37, 1.96) | 0.7049 | 1.23 (0.47, 3.19) | 0.6759 |
| Q4 | 0.68 (0.26, 1.77) | 0.4247 | 1.08 (0.39, 3.01) | 0.8840 |

Multivariable model is adjusted for age, sex, level of education, BMI, smoking history, hypertension, TC, TG and HDL
