# Supplementary material for: Dietary copper intake and risk of myocardial infarction in US adults: A propensity score-matched analysis
Source: Front Cardiovasc Med. 2022 Nov 10;9:942000. doi: 10.3389/fcvm.2022.942000 (PMC9685336; doi:10.3389/fcvm.2022.942000)
Supplement: Supplementary file 8 [file Table_8.DOC]

### **Table S8 Association between copper intake and myocardial infarction as categorized by blood lipid**

| **Subgroup** | **Before Matching** | | **After Matching** | |
| --- | --- | --- | --- | --- |
|  | **OR(95%CI)** | **P-value** | **OR(95%CI)** | **P-value** |
| **Ortholiposis** | **0.76 (0.61, 0.95)** | **0.0154** | **0.77 (0.61, 0.98)** | **0.0340** |
| Q1 | 1.0 |  | 1.0 |  |
| Q2 | 0.76 (0.56, 1.03) | 0.0747 | **0.67 (0.47, 0.96)** | **0.0284** |
| Q3 | 0.80 (0.59, 1.09) | 0.1553 | 0.88 (0.61, 1.25) | 0.4679 |
| Q4 | **0.63 (0.45, 0.88)** | **0.0075** | **0.64 (0.44, 0.94)** | **0.0226** |
| **Dyslipidemia** | 0.82 (0.66, 1.02) | 0.0685 | 0.81 (0.64, 1.02) | 0.0745 |
| Q1 | 1.0 |  | 1.0 |  |
| Q2 | 0.80 (0.57, 1.11) | 0.1797 | 0.89 (0.60, 1.34) | 0.5902 |
| Q3 | 0.76 (0.54, 1.08) | 0.1227 | 0.79 (0.52, 1.20) | 0.2658 |
| Q4 | 0.78 (0.54, 1.13) | 0.1896 | 0.76 (0.50, 1.16) | 0.2059 |
