# Supplementary material for: Dietary copper intake and risk of myocardial infarction in US adults: A propensity score-matched analysis
Source: Front Cardiovasc Med. 2022 Nov 10;9:942000. doi: 10.3389/fcvm.2022.942000 (PMC9685336; doi:10.3389/fcvm.2022.942000)
Supplement: Supplementary file 9 [file Table_9.DOC]

## Table S9 **Baseline characteristics of participants according to** IPTW

| **Variables** | **Control Group**  **(n = 14886)** | **MI Group**  **(n =11425)** | **P-value** | **SMD** |
| --- | --- | --- | --- | --- |
| **Age(years old)** | 50.17 ± 17.49 | 56.45 ± 15.21 | <0.001 | 0.383 |
| **Sex, n(%)** |  |  | 0.467 | 0.053 |
| Male | 7327 (49.22) | 5927(51.88) |  |  |
| Female | 7559 (50.78) | 5498 (48.12) |  |  |
| **Level of education, n(%)** |  |  | 0.369 | 0.143 |
| Less than 9th grade | 1089( 7.32) | 1000( 8.76) |  |  |
| 9-12th grade | 1761(11.83) | 1393(12.19) |  |  |
| High school graduate/GED or equivalent | 3375 (22.67) | 3146(27.54) |  |  |
| Some college or AA degree | 4731(31.78) | 3230(28.27) |  |  |
| College graduate or above | 3929(26.39) | 2655(23.24) |  |  |
| **BMI(kg/m^2^)** |  |  | 0.359 | 0.106 |
| ＜25 | 4035(27.11) | 3026(26.48) |  |  |
| 25-30 | 4777(32.09) | 3192(27.94) |  |  |
| ≥30 | 6074(40.80) | 5207(45.58) |  |  |
| **Smoking history,**  **n(%)** |  |  | 0.008 | 0.198 |
| No | 8355(56.12) | 5287(46.27) |  |  |
| Yes | 6531(43.88) | 6138(53.73) |  |  |
| **Drinking history, n(%)** |  |  | 0.758 | 0.023 |
| No | 4962(33.34) | 3934(34.43) |  |  |
| Yes | 9924(66.66) | 7491(65.57) |  |  |
| **Hypertension, n(%)** |  |  | <0.001 | 0.348 |
| No | 8223(55.24) | 4356(38.13) |  |  |
| Yes | 6663(44.76) | 7069(61.87) |  |  |
| [**Diabetes**](javascript:;)**, n(%)** |  |  | <0.001 | 0.318 |
| No | 10669(71.67) | 6742(59.01) |  |  |
| Yes | 2960(19.88) | 3858(33.77) |  |  |
| IGT+IFG | 1258( 8.45) | 825( 7.22) |  |  |
| **TC(mmol/L)** | 4.94 ± 1.09 | 5.04 ± 1.47 | 0.456 | 0.077 |
| **TG(mmol/L)** | 1.72 ± 1.54 | 1.77 ± 1.15 | 0.468 | 0.036 |
| **HDL(mmol/L)** | 1.37 ± 0.41 | 1.35 ± 0.47 | 0.547 | 0.047 |
| **Copper intake quartiles(%)** |  |  | 0.299 | 0.138 |
| Q1（0.0655-0.807） | 3710(24.92) | 3486(30.51) |  |  |
| Q2（0.807-1.082） | 3732(25.07) | 2554(22.36) |  |  |
| Q3（1.082-1.44） | 3730(25.06) | 2494(21.83) |  |  |
| Q4（1.44-10.6205） | 3715(24.95) | 2890(25.30) |  |  |
