# Supplementary material for: Dietary copper intake and risk of myocardial infarction in US adults: A propensity score-matched analysis
Source: Front Cardiovasc Med. 2022 Nov 10;9:942000. doi: 10.3389/fcvm.2022.942000 (PMC9685336; doi:10.3389/fcvm.2022.942000)
Supplement: Supplementary file 10 [file Table_10.DOC]

## Table S10 Correlation analysis between copper intake and myocardial infarction according to IPTW

|  | **Model 1**  **β（95% CI）**  **p value** | **Model 2**  **β（95% CI）**  **p value** | **Model 3**  **β（95% CI）**  **p value** |
| --- | --- | --- | --- |
| **Copper** | 0.78(0.73,0.82) <0.0001 | 0.82(0.77,0.87) <0.0001 | 0.85(0.80,0.91) <0.0001 |
| **Copper(mg/d) quartiles** |  |  |  |
| Q1（0.0655-0.807） | 1.0 | 1.0 | 1.0 |
| Q2（0.807-1.082） | 0.77(0.70,0.84) <0.0001 | 0.70(0.63,0.77) <0.0001 | 0.72(0.65,0.79) <0.0001 |
| Q3（1.082-1.44） | 0.79(0.73,0.87) <0.0001 | 0.75(0.68,0.82) <0.0001 | 0.79(0.72,0.88) <0.0001 |
| Q4（1.44-10.6205） | 0.65(0.59,0.71) <0.0001 | 0.68(0.61,0.75) <0.0001 | 0.73(0.66,0.81) <0.0001 |

## Model 1: No adjustments made for confounding factors

## Model 2: Adjustments made for age, sex, level of education and BMI Model 3: Adjustments same as that in model 2 plus smoking history, hypertension, diabetes, TC, TG, HDL
