# Supplementary figures and images for: Dietary copper intake and risk of myocardial infarction in US adults: A propensity score-matched analysis
Source: Front Cardiovasc Med. 2022 Nov 10;9:942000. doi: 10.3389/fcvm.2022.942000 (PMC9685336; doi:10.3389/fcvm.2022.942000)

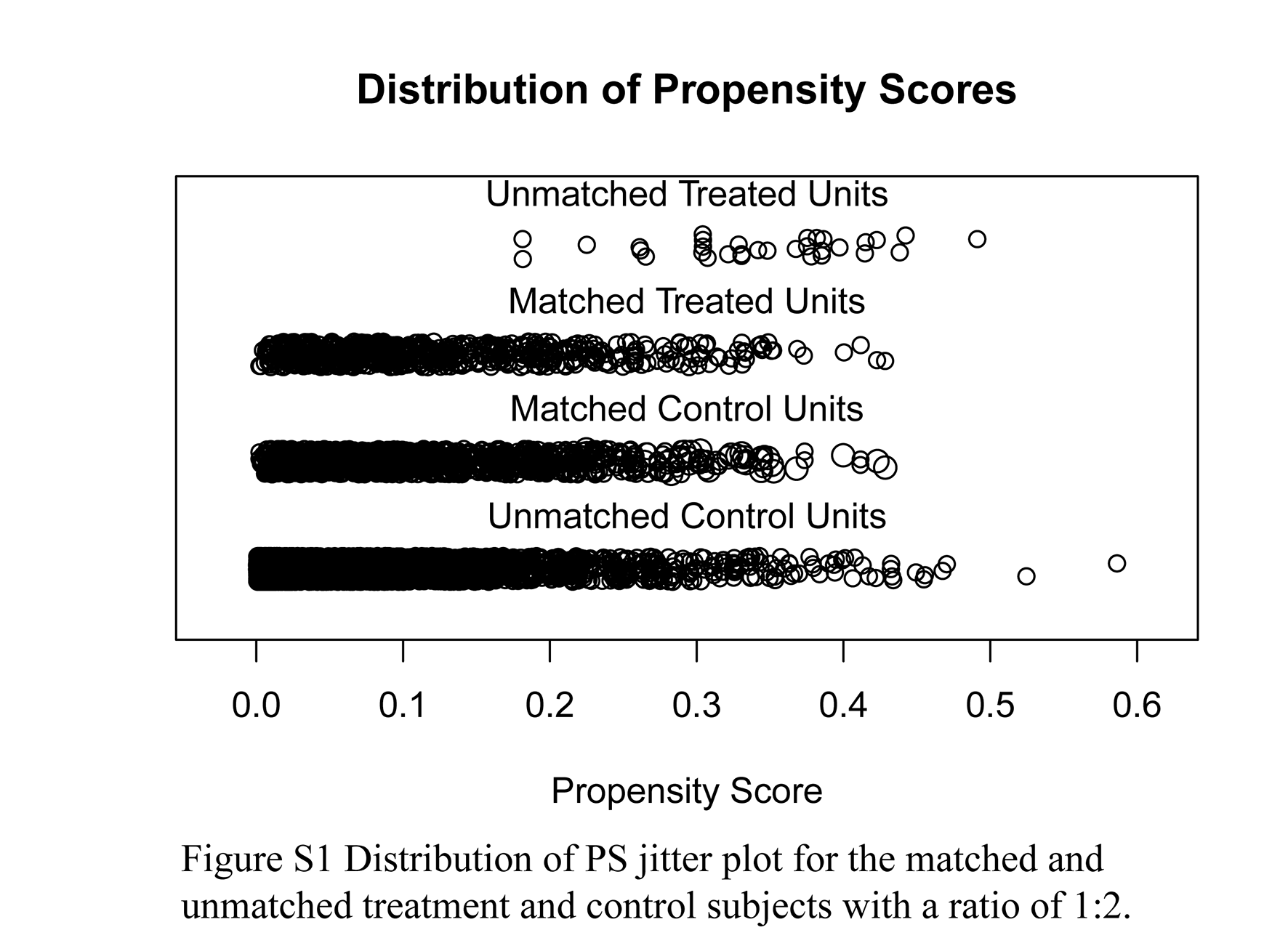

Supplement: Supplementary file 11 [file Image_1.TIF]

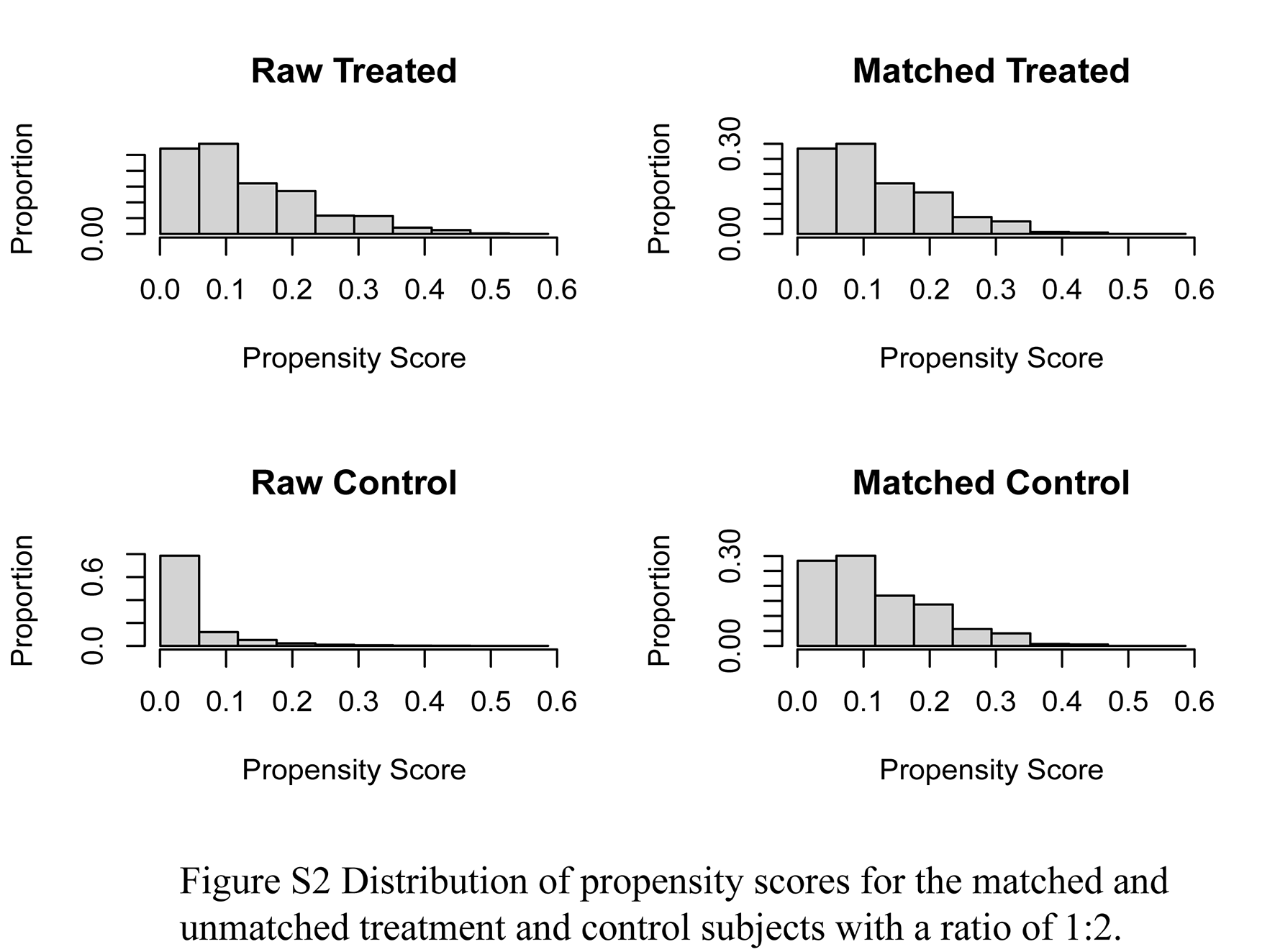

Supplement: Supplementary file 12 [file Image_2.TIF]
